# Health Tract, No. 226

**Published:** 1864-10

**Authors:** 


HEALTH TRACT, No. 226.

### SALT RHEUM

Is a disease of the blood, it is an effort of nature to push out of the system, through the skin, that which if retained would work mischief, hence any external application calculated to heal it up or drive it in, is unnatural, unwise and mischievous under all circumstances. There are states of the system in which a hasty " healing up " may be followed by long, painful and dangerous attacks of illness ; on precisely the same principle that the " striking in " of measles or any other " rash " endangers life. Hence incalculable mischief is often caused by heeding newspaper articles, such as the following : " Petroleum, crude or refined, applied thrice a day to the part affect ed with Salt Rheum, is an effectual and speedy cure." This is called a " simple " remedy, because all are familiar with the article. The Salt Rheum may disappear under such applications, but how many in a short time afterwards are attacked with violent diseases can never be known, and no inquiries are made to that effect. There is only one safe general rule as to breakings out on the skin, and that is consult the family physician at once. The next best plan is, keep warm in bed in a cool, well ventilated room, drinking warm teas into which has been broken the crust of cold wheaten bread. This is the safest, the best and most efficient course of treatment for all " breakings out " on the skin. All external applications are uncertain, worthless or injurious, as far as skin affections are concerned, except so far as they tend to keep the skin soft, moist and natural. Nothing does these things so uniformly and so well as lukewarm water, or milk and water, half and half. A little grease from a candlestick was advised to be applied to a little pimple on the child of Judge N., our neighbor, it began at once to inflame and death ensued in twenty-four hours.

